# Increased Circulating Endothelial Microparticles Associated with PAK4 Play a Key Role in Ventilation-Induced Lung Injury Process

**DOI:** 10.1155/2017/4902084

**Published:** 2017-02-05

**Authors:** Shuming Pan, Aihua Fei, Lihong Jing, Xiangyu Zhang, Chengjin Gao

**Affiliations:** ^1^Emergency Department, Xinhua Hospital Affiliated to Shanghai Jiao Tong University School of Medicine, Shanghai 200092, China; ^2^Emergency Department, The Affiliated Hospital of Qingdao University, Shandong 266100, China; ^3^Department of Critical Care Medicine, Shanghai Tenth People's Hospital, Tongji University School of Medicine, Shanghai 200072, China

## Abstract

Inappropriate mechanical ventilation (MV) can result in ventilator-induced lung injury (VILI). Probing mechanisms of VILI and searching for effective methods are current areas of research focus on VILI. The present study aimed to probe into mechanisms of endothelial microparticles (EMPs) in VILI and the protective effects of Tetramethylpyrazine (TMP) against VILI. In this study, C57BL/6 and TLR4KO mouse MV models were used to explore the function of EMPs associated with p21 activated kinases-4 (PAK-4) in VILI. Both the C57BL/6 and TLR4 KO groups were subdivided into a mechanical ventilation (MV) group, a TMP + MV group, and a control group. After four hours of high tidal volume (20 ml/kg) MV, the degree of lung injury and the protective effects of TMP were assessed. VILI inhibited the cytoskeleton-regulating protein of PAK4 and was accompanied by an increased circulating EMP level. The intercellular junction protein of *β*-catenin was also decreased accompanied by a thickening alveolar wall, increased lung W/D values, and neutrophil infiltration. TMP alleviated VILI via decreasing circulating EMPs, stabilizing intercellular junctions, and alleviating neutrophil infiltration.

## 1. Introduction

VILI is associated with increasing vascular permeability and alveolar edema and increases the expression of proinflammatory cytokines [1, 2]. Disruptions of vascular endothelial barriers are a consequence of lung inflammation and the loss of integrity of endothelial cellular barriers via intimate involvement with the cytoskeletons. Activation of the cytoskeletal apparatus results in a loss of barrier integrity and the amplification of inflammatory processes, with increased infiltration of inflammatory cells [3].

Our previous studies focusing on lung injury also indicated that disorders of cytoskeletons damaged the integrity of the pulmonary endothelial barrier, subsequently resulting in pulmonary edema and amplification of inflammation [4]. Upon activation or apoptosis, endothelial membrane microparticles (EMPs) are shed by endothelial cells associated with cytoskeletal disorders [5]. EMPs are plasma membrane vesicles <1.5 *μ*m in diameter, mainly composed of lipids and proteins [6]. Increased levels of circulating EMPs have been proposed as an aspect of cellular dysfunction [6]. We previously investigated Rho-kinase-regulated EMPs released by altering cytoskeletons [4, 7], while the detailed regulatory mechanisms were not illustrated.

p21-activated kinases (PAKs) are well known effector proteins for the Rho GTPase. PAK4, a subfamily of serine/threonine kinases originally known as a regulator of cytoskeletal dynamics and cell motility, is a group II PAK that specifically binds most strongly Rho GTPase [8]. As the effector of Rho GTPase, PAK4 controls the cytoskeleton primarily through the regulation of polymerized actin structures, particularly the formation of filopodia and lamellipodia, but can also act upon microtubule organization [9].

Toll-like receptor-4 (TLR-4) is essential to innate immune responses and has been implicated in the pathobiology of acute inflammatory lung injury [10]. Furthermore, some other studies have demonstrated that TLR4 pathway activation is also an important mechanism involved in high tidal volume induced-VILI in the absence of bacterial infection; this has increased the understanding of lung innate immunity responses, as well as the negative inflammatory effects of mechanical stress-induced lung injury [11].

Therefore, in consideration of the importance of the regulatory effects of PAK4 on cytoskeletons, maintaining normal cell shape and decreasing the release of EMPs should be a useful approach for lung protection from pulmonary injury, including VILI. We previously found that ligustrazine (Tetramethylpyrazine (TMP)) injection exerted pulmonary protective effects by reducing the release of EMPs, indicating that TMP might have modulatory effects on endothelial cytoskeletons [7, 12, 13].

In this study, we hypothesized that VILI induced EMP release by altering the cytoskeleton in which PAK4 was involved. To test this hypothesis, we used C57BL/6 and TLR4 knockout mice MV models to analyze the regulatory mechanisms of PAK4 on EMPs and further assessed the mechanisms of the TLR4 pathway in VILI. The protective effects of TMP on VILI were also determined.

## 2. Materials and Methods

### 2.1. Animal Model

Eight-week-old male C57BL/6 mice and TLR4 knockout (TLR4 KO) mice (weighing 20 ± 2 g) were used in our study and divided into the C57BL/6 group (*n* = 54) and the TLR4KO group (*n* = 54). Each mouse was anesthetized with an intraperitoneal injection of 65 mg/kg of pentobarbital sodium and intubated via tracheostomy. MV was performed with a rodent ventilator (Harvard Apparatus, Holliston, MA, USA).

Each group was subdivided into three experimental subgroups: a mechanical ventilation (MV) group (*n* = 24), in which the mice were ventilated with high tidal volume (VT = 20 ml/kg) for 4 hours; a ligustrazine (Tetramethylpyrazine (TMP)) + MV group (*n* = 24), in which the mice were intraperitoneally injected with 80 mg/kg TMP 30 minutes before the mechanical ventilation with high tidal volume (VT = 20 ml/kg) for 4 hours; and a control group (*n* = 6), in which the mice underwent tracheotomy and were ventilated with low tidal volume (VT = 6 ml/kg) for 4 hours.

In each group, six animals were used for EMP detection and six for histology, immunofluorescence, and Western blot. Six were used for selectins detection, and another six were used for neutrophil detection.

Experimental animal protocols were performed in accordance with guidelines approved by the Institutional Animal Care and Use Committee of Shanghai Jiao Tong University, School of Medicine.

### 2.2. Antibodies

We used specific antibodies against PAK4 (Santa Cruz Biotechnology, California, USA), *β*-catenin (Cell Signaling Technology Inc., Massachusetts, USA), and CXCL1 (R&D Systems, Minnesota, USA).

### 2.3. Pulmonary Function Assessment by Measurement of Lung Wet-to-Dry (W/D) Ratios and Measurement of Alveolar Wall Thickness

After four hours of MV and subsequent blood collection, the two lungs harvested from each animal were separated. The right was deposited for subsequent tests (Western blot). The left was homogenized, and the homogenate was weighed. The homogenate was centrifuged (14,000*g*, 10 min) and then desiccated in an oven (70°C for 24 h) for determination of dry weight. The lung wet-to-dry weight ratio (W/D) was computed from lung wet and dry weights.

HE staining was performed on every slice section. Five portions of every section were randomly selected and analyzed with a Spot Advanced Computer Photo Analysis Microsoft System (Silicon Graphic Inc., USA) to measure the thickness of the alveolar wall. An average value and its standard deviation were calculated.

### 2.4. Immunofluorescence and Confocal Microscopy

Lung tissue from each mouse was removed after perfusion with cold PBS and 2% paraformaldehyde. Tissue was fixed in 2% paraformaldehyde for 2 h followed by cryopreservation. Lung sections (6 *μ*m) were permeabilized with 0.2% Triton X-100 for 20 min and stained as described previously [14]. The protein levels of PAK4 were determined by using the relative antibodies (1 : 200, Calbiochem). Images were taken from six random fields/sections with a confocal fluorescence microscope (Axiovert 100, LSM 510, Zeiss, Germany). Imaging conditions were maintained at identical settings with original gating performed using the negative control (no primary antibody).

### 2.5. Western Blotting Analysis

The lung tissues, perfused with Hanks' balanced solution, were removed from the animals, hand minced, mixed with 1% SDS in PBS containing protease inhibitor, and then homogenized (3 × 30 s on ice) using a Polytron (Westbury, NY). The tissue lysate was cleared by centrifugation (15,000*g*, 30 min) in a Beckman Avanti Centrifuge J-25 I using a JA-25.50 rotor. The subsequent procedures were carried out according to our previous study [15]. The primary antibodies we used were as follows: PAK4 (1 : 1000), *β*-catenin (1 : 1000), and CXCL1 (1 : 1000).

### 2.6. EMP Analyses

Blood samples were drawn from the heart apexes, and 0.5 ml of blood was obtained from each mouse. Circulating EMPs were isolated as previously described [6]. Blood cells were removed by centrifugation two times at 2,500 ×g for 15 minutes each to obtain platelet-free plasma (PFP). EMPs in the blood were measured by flow cytometry (BD FACSCanto II; BD Biosciences, San Jose, Calif) according to a previous study [16]. Subsequently, the cell-free supernatant was removed, and the PFP (platelet counts were less than measurement sensitivity; 5 × 10^3^/*μ*L) was carefully collected. Fifty microliters (50 *μ*L) of platelet-poor plasma was mixed with 50 *μ*L of a fivefold dilution of Binding Buffer (BD Biosciences) and 20 *μ*L of heparin (Novo-Heparin, 5,000 units/5 mL for injection; Mochida Pharmaceutical Co., Tokyo, Japan). Then, 5 *μ*L of Annexin V, PE/Cy5 anti-CD41 (GPIIb, clone HIP8, IgG1, BioLegend), FITC anti-CD31 (PECAM, clone, IgG1. R&D), PE anti-CD51/CD61 (integrin avß3, clone 23C6, IgG1, BD), PE anti-CD144 (VE-Cadherin, clone 123413, IgG2B), FITC anti-CD54 (ICAM-1, clone B-H17, IgG1, Abcam), and APC anti-CD62E (E-Selectin, clone HAE-1f, IgG1, BioLegend) were added and gently vortexed at room temperature for 30 min in the dark. EMPs that expressed phosphatidylserine were labeled using fluorescein-conjugated Annexin V solution (Roche Diagnostics, Mannheim, Germany) in the presence of CaCl2 (5 mM) according to the recommendation of the supplier.

EMPs were defined as elements <1.5 *μ*m and >0.1 *μ*m and were positively labeled by specific antibodies. Endothelial microparticles were defined as events detected by Annexin V+/CD62E+ or Annexin V+/CD54+ particles smaller than 1.5 *μ*m produced via activating stimuli and detected as CD144+, CD31+/CD41−, or CD51+/CD41− particles <1.5 *μ*m. We excluded high-intensity signals caused by antibody aggregation from the flow cytometry analysis.

### 2.7. Measurements of Neutrophil Counts in Bronchoalveolar Lavage Fluid (BALF)

The presence of inflammation and lung injury was determined by measuring neutrophil counts in bronchoalveolar lavage fluid (BALF) after MV and TMP + MV treatment. A tracheal cannula was inserted into the primary bronchus, and BALF was performed through the cannula by using 1 mL of ice-cold Ca2+/Mg2+-free PBS. Approximately 800 *µ*L BALF was acquired from each mouse; 180 *µ*l of BALF was applied to Thermo Scientific Cytospin 4 (Thermo Fisher Scientific, Kalamazoo, MI) at 1000 rpm for 5 minutes, followed with Wright staining and counting of the numbers of neutrophils.

### 2.8. Quantification of Soluble Endothelial-Adhesion Molecules

Plasma and lung homogenates were assayed to evaluate the level of VCAM-1, E-selectin, and P-selectin using Fluorokine® MultiAnalyte Profiling kits and a Luminex® Bioanalyzer (R&D Systems, Oxford, UK) according to the manufacturer's instructions.

### 2.9. Statistical Analysis

Data are expressed as the mean +/− standard error (SE) and were statistically analyzed using SPSS version 16.0 software (SPSS Inc., USA). The Student–Newman–Keuls test, Student's *t*-test (between two groups), or a two-way analysis of variance (ANOVA) (>2 groups) was performed. Values of *P* < 0.05 were declared statistically significant.

## 3. Results

### 3.1. TMP Alleviated Pulmonary Edema Induced by VILI

**Figure 1:**
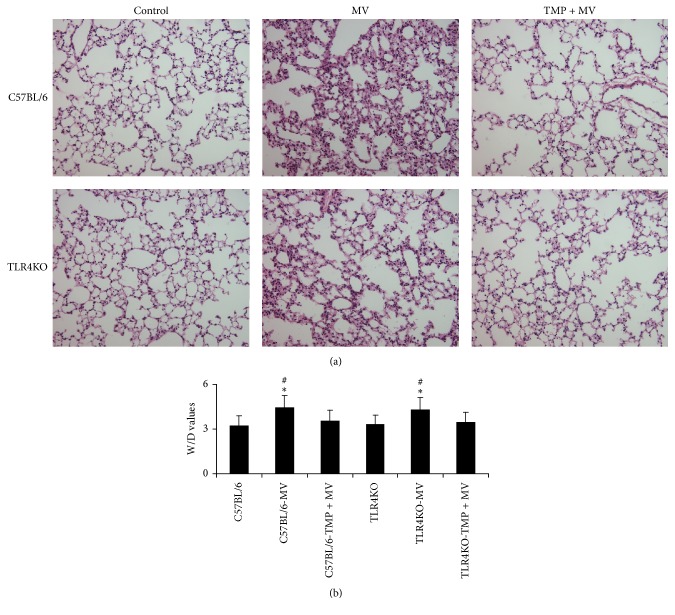
Pulmonary edema induced by VILI. (a). Hematoxylin-eosin (HE) staining of lung tissues. In both C57BL/6 and TLR4KO mice, the alveolar wall was thickened after mechanical ventilation (MV), while Tetramethylpyrazine (TMP) alleviated the thickening process. (b). Wet/dry (W/D) values of lung homogenates. In both C57BL/6 and TLR4KO mice, MV enhanced the W/D values, while TMP alleviated the enhancement. Differences are shown between mechanical ventilation- (MV-) treated mice and Tetramethylpyrazine + mechanical ventilation- (TMP + MV-) treated mice in the same group. ^*∗*^*P* < 0.05 versus the control mice of the same strain. ^#^*P* < 0.05 versus the TMP + MV-treated mice of the same strain. In both C57BL/6 and TLR4KO mice, MV resulted in pulmonary edema and TMP could alleviate the edema process.

Figure 1(a) shows the alveolar wall thickness. There was no difference in alveolar wall thickness between the C57BL/6 control mice and the TLR4KO control mice (0.94 ± 0.05 *μ*m versus 0.92 ± 0.05 *μ*m). After four hours of mechanical ventilation, the mean alveolar wall thicknesses of the C57BL/6 mice and the TLR4KO mice were 2.12 ± 0.39 *μ*m and 2.07 ± 0.35 *μ*m, respectively, while for the TMP + MV-treated mice, the mean alveolar wall thicknesses were 1.33 ± 0.14 *μ*m and 1.24 ± 0.2 *μ*m. The alveolar walls of MV-treated C57BL/6 mice were much thicker than those of the control mice and the TMP + MV-treated C57BL/6 mice (*P* < 0.05). The alveolar walls of the MV-treated TLR4KO mice were much thicker than those of the control mice and the TMP + MV-treated TLR4KO mice (*P* < 0.05, resp.).

Figure 1(b) shows the W/D values, in both C57BL/6 and TLR4KO mice; MV enhanced the W/D values, while TMP alleviated the enhancement.

### 3.2. VILI Inhibited the Protein Levels of PAK4 and *β*-Catenin

**Figure 2:**
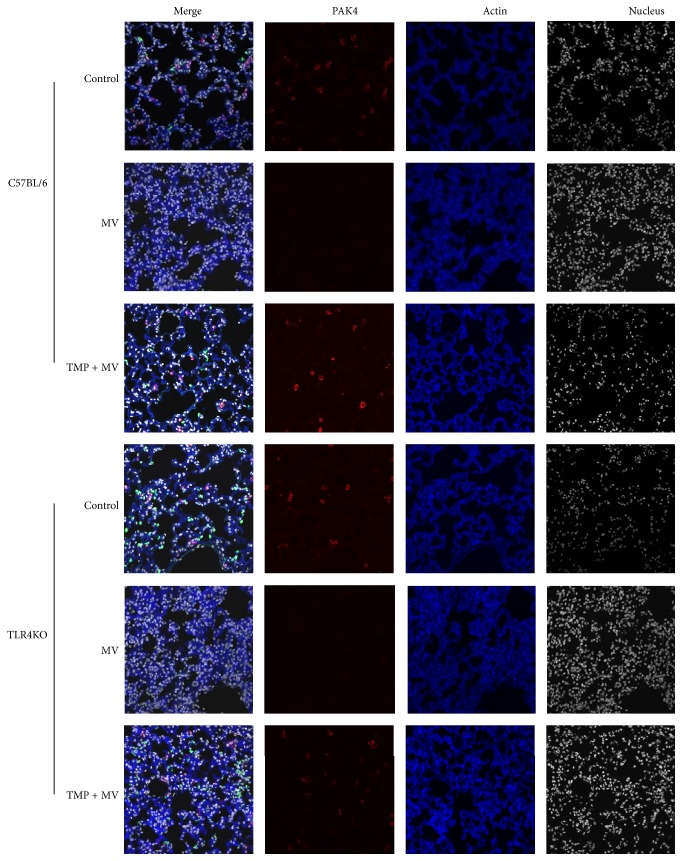
Immunofluorescence images of p21 activated kinase-4 (PAK-4) of lung tissues. Red: PAK4. Blue: actin.

**Figure 3:**
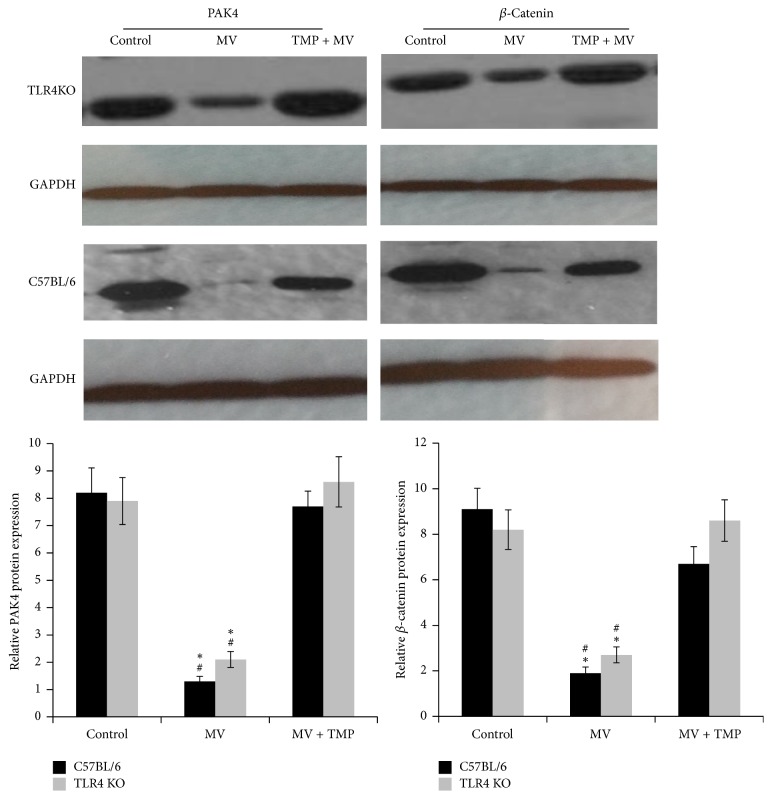
VILI decreased the pulmonary expression of CXCL1 and TMP reverted the decrease. Western blot analysis of p21 activated kinase-4 (PAK-4) and *β*-catenin, and quantification of blots to GAPDH. ^#^*P* < 0.05 versus the control mice. ^*∗*^*P* < 0.05 versus the TMP + MV-treated mice.

We compared the expressions of PAK4 and *β*-catenin in the lung tissues of the control animals, MV-treated animals, and TMP + MV-treated animals via immunofluorescence. Figures 2(a)–2(f) show the protein fluorescence of the two proteins in lung tissues. The PAK relative protein expressions were as follows: 7.78 ± 1.24 (C57BL/6 mice), 6.84 ± 1.07 (TLR4KO mice), 3.82 ± 0.72 (MV-treated C57BL/6 mice), 3.67 ± 0.71 (MV-treated TLR4KO mice), 6.53 ± 1.12 (TMP + MV-treated C57BL/6 mice), and 6.36 ± 1.08 (TMP + MV-treated TLR4KO mice). There was no difference in the PAK4 relative protein level between the C57BL/6 and the TLR4KO mice with the same treatment. Compared with the control mice, MV decreased the PAK4 expression (*P* < 0.05). The PAK4 expression of the TMP + MV-treated mice was much higher than that of both the control mice and the MV-treated mice (*P* < 0.05 and *P* < 0.01, resp.). The Western blot results, shown in Figure 3, demonstrated the same trend for PAK4. We also assessed the relative protein level of *β*-catenin. There was no difference in the *β*-catenin relative protein level between the C57BL/6 and TLR4 KO mice with the same treatment. Compared with the control mice, MV decreased the *β*-catenin expression (*P* < 0.05). The *β*-catenin expression of the TMP + MV-treated mice was much higher than the *β*-catenin expression of the MV-treated mice (*P* < 0.05).

### 3.3. VILI Increased the Plasma Level of EMPs

**Figure 4:**
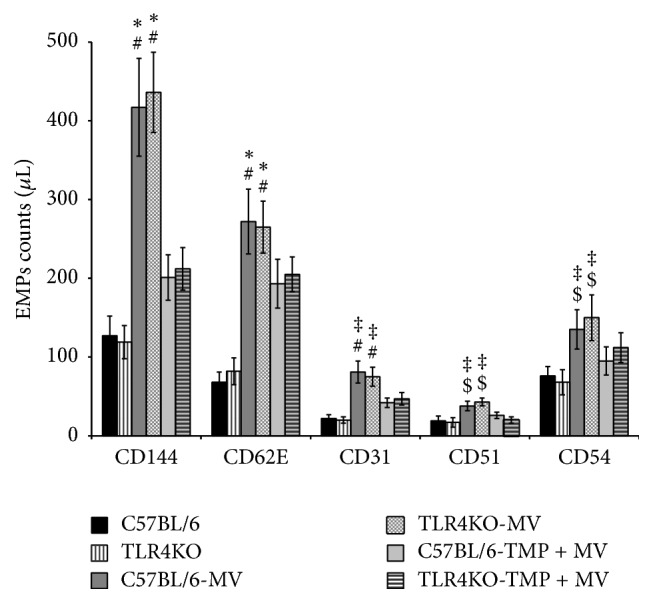
Mechanical ventilation (MV) induced endothelial microparticle (EMP) release in vivo, and Tetramethylpyrazine (TMP) decreased the MV-induced enhancement of EMPs. ^#^*P* < 0.01 versus the control mice of the same strain. ^*∗*^*P* < 0.01 versus the Tetramethylpyrazine + mechanical ventilation- (TMP + MV-) treated mice of the same strain. ^‡^*P* < 0.05 versus the TMP + MV-treated mice of the same strain. ^$^*P* < 0.05 versus the control mice of the same strain.

Figure 4 shows the EMP counts with different phenotypes. After four hours of low-tide MV, plasma EMP levels were significantly increased in both the C57BL/6 and TLR4KO mice (*P* < 0.01 for CD144, *P* < 0.01 for CD62E, *P* < 0.01 for CD31, *P* < 0.05 for CD51, and *P* < 0.05 for CD54). However, compared with the MV group, the EMP amplification of the TMP + MV-treated group was lessened (*P* < 0.01 for CD144, *P* < 0.01 for CD62E, *P* < 0.05 for CD31, *P* < 0.05 for CD51, and *P* < 0.05 for CD54).

There was no difference in the plasma EMPs level between the C57BL/6 and TLR4KO mice in the same group.

### 3.4. VILI Exaggerated the Pulmonary Inflammatory Response

#### 3.4.1. VILI Upregulated the Pulmonary Expression of CXCL1

**Figure 5:**
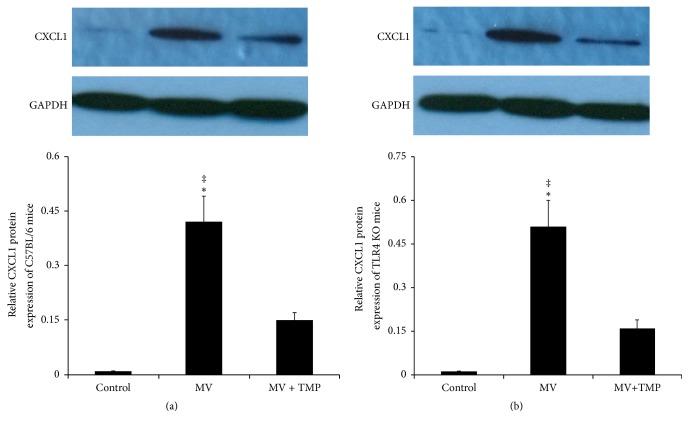
VILI upregulated the pulmonary expression of CXCL1 and TMP alleviated the upregulation. Western blot analysis of chemokine (C-X-C motif) ligand 1 (CXCL1), and quantification of blots to GAPDH. (a) C57BL/6 mice. (b) TLR4KO mice. There was no difference between the two strains of mice undergoing the same treatment. ^‡^*P* < 0.01 versus the control mice. ^*∗*^*P* < 0.05 versus the TMP + MV-treated mice.

Figure 5(a) shows the CXCL1 protein level of C57BL/6 mice lung tissues. The relative CXCL1 protein level of the control mice was 0.009 ± 0.001. After four hours of MV, the CXCL1 protein level was significantly increased (0.42 ± 0.07, *P* < 0.01). TMP treatment alleviated the enhancement. The CXCL1 protein level of the TMP + MV-treated mice (0.15 ± 0.02) was much lower than that of the MV-treated mice (*P* < 0.05). The variation trend of CXCL1 in TLR4 KO mice lung tissues was similar to that of C57BL/6 mice (Figure 5(b)). There was no difference between the two strains of mice undergoing the same treatment.

#### 3.4.2. VILI Enhanced the Neutrophils Number in Bronchoalveolar Lavage Fluid (BALF)

**Figure 6:**
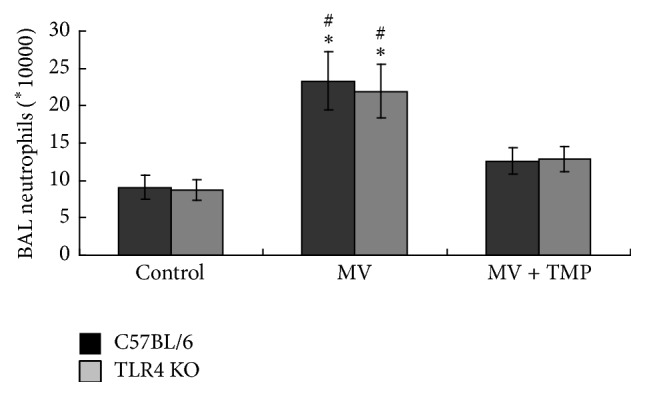
VILI enhanced the neutrophils number in bronchoalveolar lavage fluid (BALF) and TMP alleviated the filtration of neutrophils. ^*∗*^*P* < 0.05 versus the control mice. ^#^*P* < 0.05 versus the TMP + MV-treated mice of the same strain.

The neutrophil counts in the BALF are shown in Figure 6. They were significantly higher in the MV-treated C57BL/6 mice than in both the control C57BL/6 mice and the TMP + MV-treated C57BL/6 mice (*P* < 0.05). The variation trend of the neutrophils in the BALF of the TLR4 KO mice was similar to that of the neutrophils of the C57BL/6 mice. There was no difference in BALF neutrophils between the two strains of mice undergoing the same treatment.

#### 3.4.3. VILI Enhanced Selectins and Adhesion Molecules Levels in Both Lungs and Serum

An ELISA assay was performed to analyze the lung and serum levels of adhesion molecules such as E- and P-selectin and VCAM-1 (Table 1). Both the C57BL/6 and TLR4KO mice after MV had a significantly higher level of serous E-selectin than the control mice and MV + TMP-treated mice. Both the C57BL/6 and TLR4KO mice had a significantly higher level of E-selectin after MV in their lung tissues than the control mice and MV + TMP-treated mice. MV did not cause significant alteration of P-selectin in the serum of either the C57BL/6 or the TLR4KO mice. Both the C57BL/6 and TLR4KO mice had a significantly higher level of P-selectin after MV in their lung tissues than the control mice and the MV + TMP-treated mice. The C57BL/6 mice had a much higher level of plasma VCAM-1 after MV than the control mice and the MV + TM-treated mice, while MV did not cause significant alteration of plasma VCAM-1 levels in the TLR4KO mice. Both the C57BL/6 and TLR4KO mice had a significantly higher level of VCAM-1 after MV in their lung tissues than the control mice and the MV + TMP-treated mice.

## 4. Discussion

In the present study, we found that 20 ml/kg of tidal volume MV could induce evident lung injury, associated with a thickening alveolar wall and increased lung W/D values. Higher W/D values represent higher pulmonary microvascular permeability. Our previous studies demonstrated that disorders of cytoskeletons and intercellular junctions led to higher pulmonary microvascular permeability. In order to investigate the probable mechanisms of VILI-induced higher pulmonary microvascular permeability, we then focused on the effects of VILI on some regulatory proteins of cytoskeletons and intercellular junctions.

The endothelial cell (EC) barrier forms a semipermeable membrane that regulates the passage of fluid and neutrophils out of the blood and into the interstitial space. Under normal conditions, ECs maintain the stable cytoskeleton system and effective intercellular junctions to preserve normal barrier function, while many pathologic conditions can damage the stabilization of intercellular junctions, subsequently resulting in endothelial hyperpermeability. Accompanied by higher W/D values, the cytoskeleton regulator PAK4 and the intercellular junction protein of *β*-catenin were all decreased in C57BL/6 mice and TLR4KO mice, demonstrating that the two proteins were involved in the abnormities of cytoskeletons and intercellular junctions induced by VILI. Several studies provide evidence that an increase in circulating EMPs could be considered a surrogate marker of the abnormities of endothelial cytoskeletons [6]. Increased EMP levels and the alterations of PAK4 caused by VILI in our study also confirmed the abnormities of endothelial cytoskeletons.

In addition to cytoskeletal disorders, VILI also damaged intercellular junctions and subsequently caused higher pulmonary microvascular permeability. Disorders of both skeletons and intercellular junctions can enlarge intercellular gaps and simultaneously result in higher pulmonary microvascular permeability.

As a principal sort of inflammatory cells, neutrophils migrate to sites of inflammation through intercellular gaps, where they exert anti-infectious and proinflammatory effects. CXCL1 is involved in neutrophil recruitment to the lung tissues after injury [17]. In our study, VILI-induced alteration of CXCL1 in lung tissues was consistent with BALF neutrophil counts, which also demonstrated not only the chemotactic effects on neutrophil recruitment to injured tissues, but also higher pulmonary microvascular permeability.

Selectins and VCAM-1 are part of a major class of adhesion molecules known to play an important role in early inflammation stages in recruiting neutrophils to the site of inflammation. Adhesion molecules play an important role in leukocyte endothelial interactions and resulting neutrophil migration into the site of injury or infection, enlarging regional and even systemic injuries [18]. Higher levels of serumal E- and P-selectin and pulmonary VCAM-1 in the VILI mice were found, indicating a more severe pulmonary injury.

Toll-like receptors (TLRs) are a variety of pathogen pattern recognition receptors that recognize pathogen-associated molecular patterns from bacterial and other pathogens [19]. TLR4 recognizes endotoxin (lipopolysaccharide [LPS]). Based on its interaction with LPS, TLR4 may be a mediator and a modulator of endotoxin-induced inflammation and shock [20]. While TLR4 has been implicated in the pathobiology of acute inflammatory lung injury via regulation of innate immune responses to Gram-negative bacteria infection, it is not clear whether TLR4 is involved in VILI. In our study, comparing two strains of mice, we found the following positive result: The plasma VCAM-1 level was increased in the VILI C57BL/6 mice, but not in the VILI TLRKO mice. The alveolar wall thickness, W/D values, EMP counts, PAK4 protein, *β*-catenin protein, CXCL1 protein, BALF neutrophil counts, and selectin levels of the two strains of mice undergoing the same treatment were not different, likely because the short time period (four hours) of mechanical ventilation (not infection) had little effect on the TLR4 pathway.

In addition to some useful mechanical ventilation adjustments, searching for other effective methods to alleviate pulmonary injury in critically ill patients is very important. We previously used TMP, a Chinese traditional herb injection, to exert pulmonary protective effects by regulating the release of EMPs, showing the applicability of TMP in both preventive and therapeutic fields. In this study, detailed mechanisms of TMP's protective effects were illustrated by PAK4's involvement in cytoskeletal regulation.

In summary, increased plasma EMPs associated with PAK4 are involved in VILI process. TMP could alleviate VILI via decreasing circulating EMPs, stabilizing intercellular junctions and alleviating neutrophil infiltration.

## Supplementary Material

The molecular formula of tetramethylpyrazine.

## Figures and Tables

**Table 1:** E-selectin, P-selectin, and VCAM-1 in serum and lung tissues.

	Serous E-selectin		Pulmonary E-selectin		Serous P-selectin		Pulmonary P-selectin		Serous VCAM-1		Pulmonary VCAM-1	
Groups	C57BL/6 (pg/mL)	TLR4KO (pg/mL)	C57BL/6 (pg/mL)	TLR4KO (pg/mL)	C57BL/6 (pg/mL)	TLR4KO (pg/mL)	C57BL/6 (pg/mL)	TLR4KO (pg/mL)	C57BL/6 (pg/mL)	TLR4KO (pg/mL)	C57BL/6 (pg/mL)	TLR4KO (pg/mL)
Control	27.26 ± 4.06	28.51 ± 4.19	107.49 ± 17.42	112.61 ± 19.34	68.46 ± 9.68	71.42 ± 10.64	46.37 ± 6.32	41.53 ± 6.87	5.26 ± 0.88	5.10 ± 0.78	13.86 ± 2.03	12.86 ± 1.96
MV	41.43 ± 5.83^#*∗*^	44.27 ± 6.93^#*∗*^	165.34 ± 31.33^#*∗*^	171.46 ± 34.56^#*∗*^	74.36 ± 10.21	72.98 ± 11.45	68.88 ± 11.23^#*∗*^	66.26 ± 9.69^#*∗*^	8.82 ± 1.27^#^	6.26 ± 1.03	27.55 ± 5.02^#*∗*^	21.34 ± 3.46^#*∗*^
MV + TMP	33.27 ± 4.31	34.63 ± 5.12	123.45 ± 23.38	128.39 ± 27.21	70.85 ± 9.43	75.56 ± 12.37	51.32 ± 8.42	50.76 ± 7.68	6.17 ± 0.96	5.92 ± 0.92	17.46 ± 3.10	16.55 ± 3.05

Differences are shown between the two groups. Values are means for 6 mice. ^#^*P* < 0.05 versus the control mice of the same group. ^*∗*^*P* < 0.05 versus the MV + TMP mice of the same group.

## References

[1] Hoogendijk A. J., Kuipers M. T., Van Der Poll T., Schultz M. J., Wieland C. W. (2012). Cyclin-dependent kinase inhibition reduces lung damage in a mouse model of ventilator-induced lung injury. *Shock*.

[2] Woods S. J., Waite A. A. C., O’dea K. P., Halford P., Takata M., Wslson M. R. (2015). Kinetic profiling of in vivo lung cellular inflammatory responses to mechanical ventilation. *American Journal of Physiology—Lung Cellular and Molecular Physiology*.

[3] Matsumoto H., Yamakawa K., Ogura H., Koh T., Matsumoto N., Shimazu T. (2015). Enhanced expression of cell-specific surface antigens on endothelial microparticles in sepsis-induced disseminated intravascular coagulation. *Shock*.

[4] Gao C., Li R., Liu Y., Ma L., Wang S. (2012). Rho-kinase-dependent F-actin rearrangement is involved in the release of endothelial microparticles during IFN-*α*-induced endothelial cell apoptosis. *Journal of Trauma and Acute Care Surgery*.

[5] Patil R., Ghosh K., Satoskar P., Shetty S. (2013). Elevated procoagulant endothelial and tissue factor expressing microparticles in women with recurrent pregnancy loss. *PLoS ONE*.

[6] Gordon C., Gudi K., Krause A. (2011). Circulating endothelial microparticles as a measure of early lung destruction in cigarette smokers. *American Journal of Respiratory and Critical Care Medicine*.

[7] Wang H., Chen Y., Li W. (2015). Ligustrazine effect on lipopolysaccharide-induced pulmonary damage in rats. *Burns*.

[8] Zhao S., Zhang Y., Chen Q. (2015). A modified 'double-hit' induced acute lung injury model in rats and protective effects of tetramethylpyrazine on the injury via Rho/ROCK pathway. *International Journal of Clinical and Experimental Pathology*.

[9] Qu J., Li X., Novitch B. G. (2003). PAK4 kinase is essential for embryonic viability and for proper neuronal development. *Molecular and Cellular Biology*.

[10] Camp S. M., Ceco E., Evenoski C. L. (2015). Unique toll-like receptor 4 activation by NAMPT/PBEF induces NF*κ*B signaling and inflammatory lung injury. *Scientific Reports*.

[11] Huang L.-T., Lin C.-H., Chou H.-C., Chen C.-M. (2014). Ibuprofen protects ventilator-induced lung injury by downregulating Rho-kinase activity in rats. *BioMed Research International*.

[12] Gao C., Peng H., Wang S., Zhang X. (2012). Effects of Ligustrazine on pancreatic and renal damage after scald injury. *Burns*.

[13] Gao C., Liu Y., Ma L., Zhang X., Wang S. (2012). Effects of Ligustrazine on pulmonary damage in rats following scald injury. *Burns*.

[14] Lagoa C. E., Vodovotz Y., Stolz D. B. (2005). The role of hepatic type 1 plasminogen activator inhibitor (PAI-1) during murine hemorrhagic shock. *Hepatology*.

[15] Gao C., Tang J., Li R., Huan J. (2012). Specific inhibition of AQP1 water channels in human pulmonary microvascular endothelial cells by small interfering RNAs. *Journal of Trauma and Acute Care Surgery*.

[16] Matsumoto H., Yamakawa K., Ogura H., Koh T., Matsumoto N., Shimazu T. (2015). Enhanced expression of cell-specific surface antigens on endothelial microparticles in sepsis-induced disseminated intravascular coagulation. *Shock*.

[17] Hoth J. J., Wells J. D., Hiltbold E. M., McCall C. E., Yoza B. K. (2011). Mechanism of neutrophil recruitment to the lung after pulmonary contusion. *Shock*.

[18] Shah D., Romero F., Zhu Y. (2015). C1q deficiency promotes pulmonary vascular inflammation and enhances the susceptibility of the lung endothelium to injury. *Journal of Biological Chemistry*.

[19] Alloatti A., Kotsias F., Pauwels A.-M. (2015). Toll-like receptor 4 engagement on dendritic cells restrains phago-lysosome fusion and promotes cross-presentation of antigens. *Immunity*.

[20] Wu K.-H., Wu H.-P., Chao W.-R. (2016). Time-series expression of toll-like receptor 4 signaling in septic mice treated with mesenchymal stem cells. *Shock*.

