# Supplementary figures and images for: Increased Circulating Endothelial Microparticles Associated with PAK4 Play a Key Role in Ventilation-Induced Lung Injury Process
Source: Biomed Res Int. 2017 Feb 5;2017:4902084. doi: 10.1155/2017/4902084 (PMC5316431; doi:10.1155/2017/4902084)

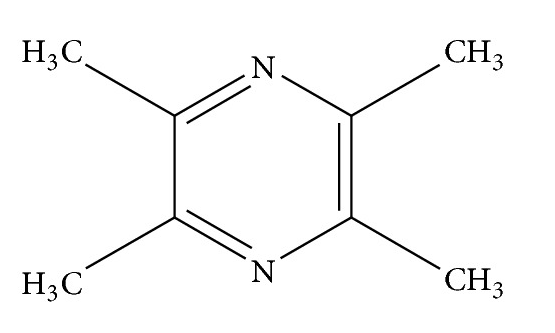

Supplement: Supplementary file 1 — The molecular formula of tetramethylpyrazine. [file 4902084.f1.tif]
